# Hypolipidemic activity of lactic acid bacteria: Adjunct therapy for potential probiotics

**DOI:** 10.1371/journal.pone.0269953

**Published:** 2022-06-23

**Authors:** Shima Mahmoud Ali, Fatma E. Salem, Mohammad M. Aboulwafa, Riham M. Shawky

**Affiliations:** 1 Faculty of Pharmacy, Department of Microbiology and Immunology, Helwan University, Ain-Helwan, Cairo, Egypt; 2 Faculty of Pharmacy, King Salman International University, Ras Sudr, South Sinai, Egypt; 3 Faculty of Pharmacy, Department of Microbiology and Immunology, Ain Shams University, Al Khalifa Al Ma’moun St., Abbassia, Cairo, Egypt; Agricultural University of Athens: Geoponiko Panepistemio Athenon, GREECE

## Abstract

**Background:**

Individuals with hyperlipidemia are two times more likely to develop atherosclerotic cardiovascular disease (ASCVD) as opposed to those with controlled serum total cholesterol (TC) levels. Considering the documented adverse events of the current lipid-lowering medications which ultimately affect patient’s compliance, substantial efforts have been made to develop new therapeutic strategies. Probiotics, on the other hand, are reported to have lipid-lowering activity with the added benefit of being generally well-tolerated making it an appealing adjuvant therapy.

**Methods:**

A total of fifty Lactic acid bacteria (LAB) were isolated from raw milk (human and animal) and dairy products. Isolates demonstrating promising *in vitro* cholesterol removal capabilities were morphologically and biochemically characterized. Lastly, two bacterial candidates were selected for evaluation of their potential hypolipidemic activity using a laboratory animal model. Statistical differences between the means were analyzed by one-way analysis of variance (ANOVA) followed by Tukey’s post-hoc test. A *p*-value < 0.05 was considered statistically significant.

**Results:**

Most of the isolates demonstrated an *in vitro* cholesterol removal activity. The six LAB isolates showing the highest cholesterol removal activity (36.5–55.6%) were morphologically and biochemically identified as *Lactobacillus*, *Pediococcus*, and *Lactococcus* species. The results demonstrated two promising antihyperlipidemic candidates, a *Lactococcus lactis* ssp. *lactis* with an *in vivo* significant reduction of serum triglycerides (TG) levels by 34.3%, and a *Pediococcus* sp. that was able to significantly reduce both the serum TC and TG levels by 17.3% and 47.0%, respectively, as compared to the diet-induced hyperlipidemic animal group.

**Conclusion:**

This study further supports the growing evidence regarding the antihyperlipidemic activity among probiotics, presenting them as a promising therapeutic approach for the management of hyperlipidemia.

## 1. Introduction

According to the World Health Organization (WHO), cardiovascular diseases (CVDs) are the primary cause of death globally. In 2019, nearly 18.6 million people lost their lives to CVD worldwide (17.1% increase from 2010) with at least 75% of the world’s CVDs -related deaths incidences occurring in low- and middle-income nations [1]. Such findings are highly alarming that non-communicable disease prevention, diagnosis, and treatment must be accelerated.

Hyperlipidemic individuals are nearly two times more likely to develop ASCVD in contrast to those with controlled serum total cholesterol levels [2]. Hyperlipidemia refers to a group of disorders characterized by elevated serum levels of cholesterol and triglycerides [3]. An increase in serum triglyceride concentrations is represented by an increase in the triglyceride-rich lipoproteins including chylomicrons, very low-density lipoprotein (VLDL-C), as well as their remnants. However, an increase in blood cholesterol levels is represented by an elevation in serum low-density lipoprotein (LDL-C) levels, with or without an increase in VLDL-C levels [4].

According to "The 3rd Report of the Expert Panel on Detection, Evaluation, and Treatment of High Blood Cholesterol in Adults"(ATP III), optimal LDL-C levels is <100 mg/dL, desirable total cholesterol level is <200 mg/dL, normal triglycerides level is <150 mg/dL and HDL-C level not below 40 mg/dL [5].

Cholesterol deposition in arterial walls ultimately leads to atherosclerosis in which narrowing, thickening, or blocking of the arterial lumen occurs and this is the hallmark of CVD [6]. TC reduction has been a vital aspect of public health strategies, and several effective pharmacological and non-pharmacological approaches have been implemented in this regard. As for the lipid-lowering medications, several pharmacokinetic interactions affect their activity and/ or toxicity. In addition, a lot of patients do not reach their lipid profile target levels using statins as monotherapy due to treatment resistance, abstinence due to drug-related adverse events, and poor compliance [7]. Consequently, many guidelines recommend an add-on therapy such as ezetimibe, fibrates, nicotinic acid, bile acid sequestrants, and PCSK9 drugs. However, these drugs have as well documented side effects and contraindications which puts further constraints regarding their use [8].

Mann and Spoerry have noticed the hypocholesterolemic activity of the fermented milk consumed by the people of the Massai tribes [9] and since then the connection between LAB and blood cholesterol levels has garnered substantial attention. Probiotics are living microorganisms that, when implemented appropriately, provide a variety of health benefits to the host. like: enhanced digestion, increased immunity, cancer suppression, and decreased serum cholesterol level [10]. The majority of probiotics are commonly referred to as LAB because they generate lactic acid as a fermentation product. According to the morphological and phenotypic characteristics, LAB was categorized into several genera that include *Lactobacillus*, *Bifidobacterium*, and *Enterococcus* sp. [11,12].

*Lactobacillus* bacteria are lactic acid-producing Gram-positive rods, obligate and/or facultative anaerobes that are mainly found in the human gastrointestinal and genitourinary tracts [13,14]. *Bifidobacterium* is also Gram-positive rods but mostly straight anaerobes, pleomorphic, non-spore-forming bacteria and they produce yield lactic and acetic acids from carbohydrate fermentation[15,16]. Both *Lactobacilli* and *Bifidobacteria* have shown hypocholesterolemic effects in both animal models[17–19] and humans [20–23]. Additionally, several hypotheses were put to explain the mechanism by which probiotics produce their hypolipidemic activity [24–27]. Other studies, however, are paradoxical and fail to prove probiotics’ hypolipidemic effects [28,29]. As a result, this subject remains a controversial issue and more research is needed [30].

Although basic and clinical studies on probiotics have evolved rapidly, probiotics still are not applied as drugs on a worldwide platform [31]. Yet, under the supervision of the US Food and Drug Administration (FDA), clinical physicians in America have proposed probiotics as evidence-based medical foods and dietary supplements [32]. Several probiotics are "Generally Recognized as Safe" (GRAS) for use in foods. Furthermore, the European Food Safety Authority (EFSA) ascertained that a number of traditional probiotic strains were safe for use in food through using "Qualified Presumption of Safety" (QPS) [33]. Interestingly, from October 2019 till March 2020, EFSA discovered that only six microorganisms out of 39 reports fit the criteria [34]. However, these strains are insufficient to meet the diverse needs of industry and humans [35]. Consequently, additional strains of bacteria must be discovered in order to broaden the application panel of probiotics and their implementations in the food industry [32]. Therefore, this study aimed to screen for a probiotic species with potential anti-hyperlipidemic activity from raw milk (human and animal) and dairy products.

## 2. Methods

### 2.1. Samples collection

#### 2.1.1. Breast milk

A total of 25 Samples of breast milk were obtained from Egyptian lactating females attending the outpatient pediatric and/or obstetrics and gynecology clinics at different local hospitals. Subjects were chosen and milk samples were collected according to the criteria and procedures described by [36] with a slight modification of swabbing the areola with an alcohol swab before sample collection. The study was approved by the Ethics Committee of the Faculty of Pharmacy, Helwan University, and prior to their enrollment in the study, the mothers were informed of the investigational character of the study. The participant’s consent was obtained verbally. No minors were included in the study.

#### 2.1.2. Raw animal milk and dairy products

A total of twenty-five samples were obtained from dairy farms in Cairo, Egypt, and traditional commercial Egyptian dairy products (milk, cheese, yogurt, and cream).

All samples, the breast milk, raw animal milk, and dairy products, were transferred in sterile plastic bottles to the laboratory and immediately used for bacterial isolation.

### 2.2. Recovery of LAB

The pour plate technique was used to isolate the microorganisms [36]. For milk samples, 1 mL milk was inoculated into 9 mL of sterile saline solution (S.S.S) whereas, for the solid dairy products, 10 grams were aseptically homogenized in 90 mL of S.S.S. Samples were serially diluted (10 fold) then 1 mL aliquot of each dilution was plated into 9 mL of molten De Man, Rogosa, and Sharpe (MRS) medium agar (Sigma-Aldrich). Inoculated MRS plates were incubated anaerobically at 37°C for 48–72 hrs. in an anaerobic jar (GasPak System—Oxoid, Basingstoke Hampshire, England) using AnaeroPack™-Anaero anaerobic gas generator sachets (Mitsubishi, Japan). Typical colonies were selected according to the morphological characters described by [37], picked from the plates, and repeatedly sub-cultured to obtain pure isolates.

### 2.3. Maintenance and preservation of isolates

The different isolates were preserved in an MRS broth medium containing glycerol (20%) and stored at -80°C until further testing.

### 2.4. Testing the cholesterol removal activity of LAB isolates

Before each assay, the isolates were activated three times by successive sub-culturing in an MRS broth. The potential of the working isolates to remove cholesterol from the medium was assessed according to [38] with minor modification. Briefly, to prepare the cholesterol stock solution, 10 mg of cholesterol were dissolved in 1 mL of 96% ethyl alcohol [39]. Cholesterol was filter sterilized (0.20 μm, Genetix Biotech Asia, New Delhi, India) and added to the sterile MRS broth at a final concentration of approximately100 mg/L. The freshly cultured bacterial isolates were inoculated into the sterile MRS broth at 1% (v/v) inoculum size and incubated anaerobically at 37°C for 18 hr. The un-inoculated broth was used as a control. Lastly, cultures were centrifuged at 10,000 g for 15 min at 4°C, and the resultant supernatants were taken to determine the residual cholesterol concentrations by capillary gas chromatography [40]. The experiment was repeated two more times for the isolates showing the highest cholesterol removal activity and they were selected for the following investigations.

### 2.5. Characterization and identification of the selected isolates

#### 2.5.1 Morphological characterization

Microscopic examination following the Gram staining technique was used in accordance with [41] to identify the cell shape and arrangement of the isolates that demonstrated the highest cholesterol removal activity.

#### 2.5.2. Biochemical characterization

The catalase test was carried out following the procedures described in [42]. API 50 CH test kit (BioMerieux, Marcy L’Etoile, France) in combination with 50 CHL liquid media were used to determine the carbohydrate fermentation patterns of the selected isolates [43].

### 2.6. Animal feeding trial: Testing the effect of two selected LAB isolates on serum lipid levels in a laboratory animal model

#### 2.6.1. Inoculum preparation

Two LAB isolates out of the two selected genera (*Pediococcus* and *Lactococcus*) that showed the highest and most reproducible effect on cholesterol removal *in vitro* were used for the animal feeding trial. A standard calibration curve between colony-forming units (CFU) and their corresponding optical densities (O.D) for each test isolate was constructed [44]. Briefly, an overnight culture of each test isolate was prepared using a single colony from an 18hrs fresh agar culture, followed by a sequential decimal dilution in S.S.S. The O.D and the viable count of each resultant dilution were recorded. The viable count was carried out by plating 100 μL of the appropriate dilution in MRS-agar plates. Plates were incubated for 18hrs at 37°C under anaerobic conditions. The experiment was carried out 3 times, and the data obtained were averaged and a curve of O.D against CFU/mL was plotted (S7 Table (Supporting Information)). Lastly, the O.D of the two test isolates was adjusted to the corresponding required viable count of 1 x 10^9^ CFU/mL for the animal feeding experiment [38] using the resultant equation *y = mx + c*, where "*m"* is the gradient and *"c*" is the y-intercept.

#### 2.6.2. Preparation of atorvastatin

For humans, the initial treatment dosage of atorvastatin (Lipitor, Pfizer) is ten mg daily. The hamster’s dose was determined by utilizing the human equivalent dose (HED) and body surface area (BSA). In brief, human daily dose per kilogram multiplied by the variation coefficient of the BSA between humans and hamsters [45,46]:

10 mg (human daily dose) / 60 kg (average human body weight) X 7.4 (variation coefficient in BSA) = 1.23 mg/kg.

#### 2.6.3. Animal and study design

All animal studies were carried out following the Guide for the Care and Use of Laboratory Animals [47]. The procedures involving animals were approved by The Animal Ethics Committee of Helwan University. The study was designed following the procedures described in [38], with some modifications. Briefly, six-week-old male Golden Syrian hamsters were obtained from Theodor-Bilharz Research Institute (TBRI), Egypt. Hamsters were accommodated in a room with a humidity of 60±5%, a temperature of 22±2°C, a 12-hour light-dark cycle, and a standard food diet (Std). For the induction of hyperlipidemia, the animals were fed a high-fat diet (HFD) which was designed in accordance with the Paigen atherogenic diet [48].

After 1 week of acclimation, animals were randomly divided into five groups (n = 6). The first group (non-induced) was fed a standard food diet while the second group (diet-induced hyperlipidemia) was fed the HFD, these two groups received saline by oral gavage daily. The third group received the HFD + 1 × 10^9^ CFU/mL/day of *Lactococcus lactis* ssp. *lactis*. The fourth group received the HFD + 1 × 10^9^ CFU/mL/day of *Pediococcus* sp. The fifth group received the HFD + 1.23 mg/kg/day atorvastatin used as a positive control. During the feeding period, all animals had free access to water and their assigned food regime. The animals were kept on the previously stated regime for 28 days.

### 2.7. Measurement of serum lipid levels in tested laboratory animals

At the end of the twenty-eight days of the feeding trial, all tested hamsters were kept fasting for 12 hours then sacrificed. The whole blood was collected from the retro-orbital plexus then the serum was obtained by centrifugation at 3000 g for 15 minutes. The enzymatic colorimetric method and an AU680 automated biochemical analyzer (Beckman Coulter, Fullerton, CA, USA) were used for the determination of serum TC, LDL-C, HDL-C, and TG levels.

### 2.8. Statistical analysis

Data were expressed as mean ± SEM and the analysis was performed using SPSS software (version 22.0, IBM Corporation, Armonk, NY, USA). Statistical differences between the means were analyzed by one-way analysis of variance followed by Tukey’s post-hoc test. A *p*-value < 0.05 was considered statistically significant.

## 3. Results

### 3.1. Removal of cholesterol by the recovered LAB isolates

**Fig 1:**
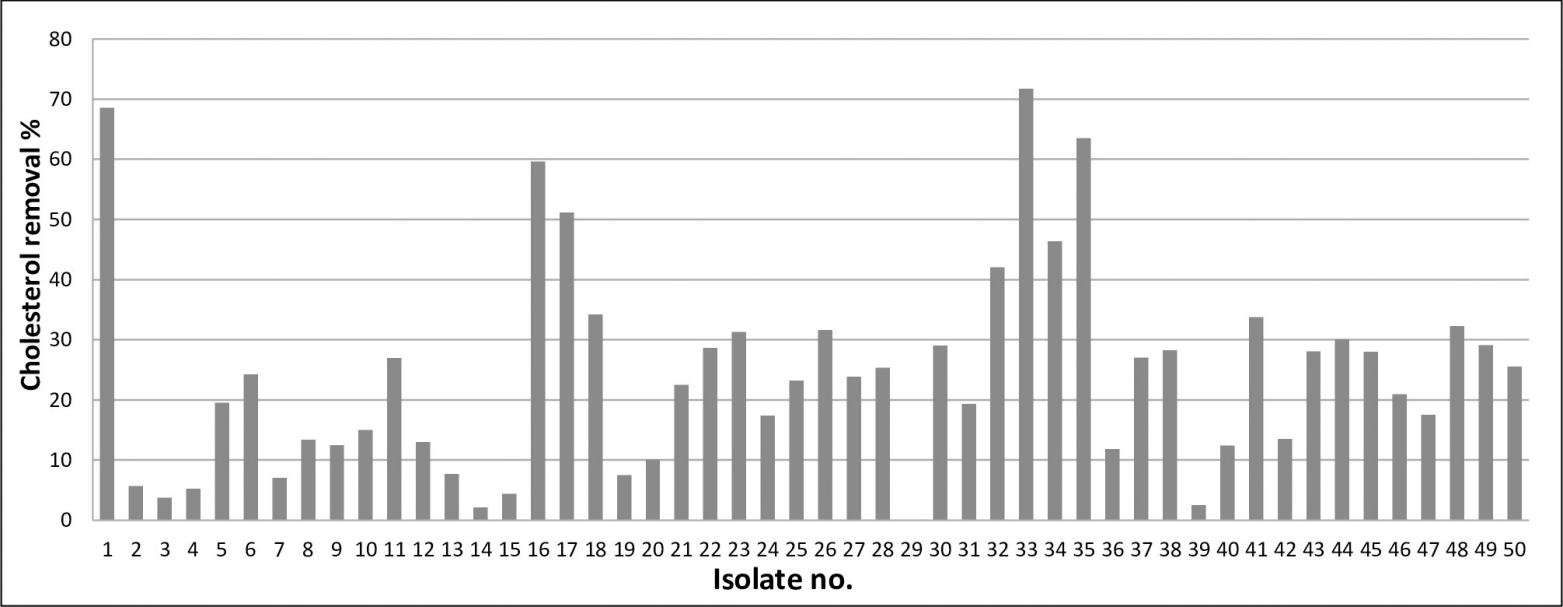
Preliminary screening of LAB isolates for their cholesterol removal capability.

A total of 50 LAB were recovered from raw (human and animal) milk and other relevant dairy products in this study and analyzed *in vitro* for their cholesterol removal ability. Forty-nine out of 50 tested bacterial isolates were able to remove cholesterol from the fermentation broth during the 18 hours incubation period. The results are shown in Fig 1 and S1 Table (Supporting Information).

**Fig 2:**
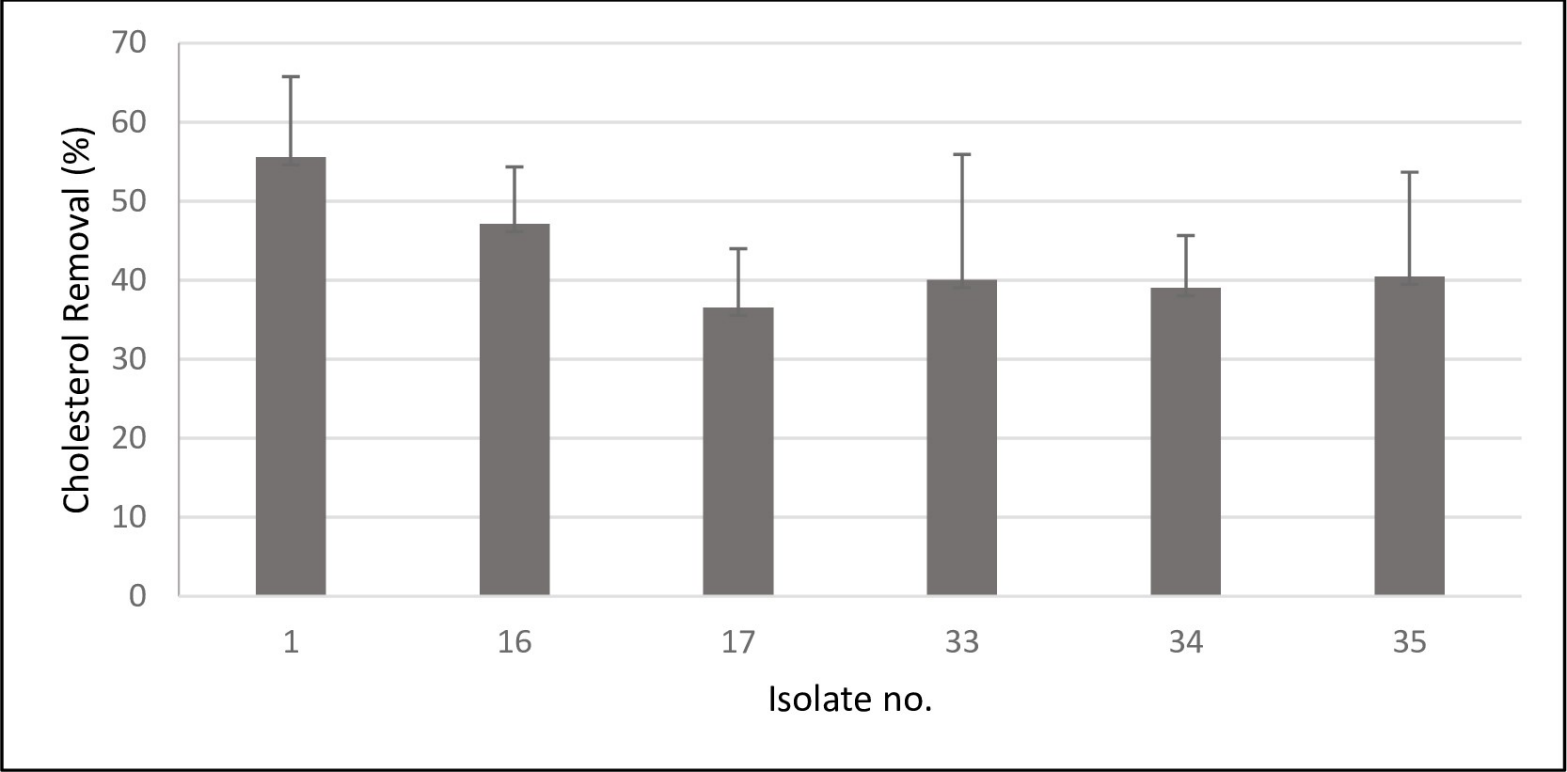
Comparison of cholesterol removal capabilities of six selected LAB isolates showing high activity in preliminary screening. Data are represented as the mean ±SEM (n = 3).

The cholesterol removal percentages varied among the LAB isolates, ranging from 2.2% to 71.7%. Isolate no.29 had no cholesterol removal ability (0.05%). The isolates that exhibited the highest cholesterol removal ability were of the following numbers: 1, 16, 17, 33, 34, and 35 with cholesterol removal percentages values of 68.6%, 59.6%, 51.2%, 71.7%, 46.4%, and 63.5%, respectively. These isolates were checked for their reproducible effect by repeating the same experiment two more times and the results are shown in Fig 2 and S2 Table (Supporting Information).

The order of cholesterol removal capabilities of the six tested isolates were as follow: isolate no. 1 (55.6% ±SEM = 10.166) > isolate no.16 (47.1% ±SEM = 7.205) > isolate no. 35 (40.5% ±SEM = 13.2) > isolate no.33 (40.0% ±SEM = 15.88) > isolate no. 34 (39.0% ±SEM = 6.6) > isolate no. 17 (36.5% ±SEM = 7.4).

### 3.2. Characterization and identification of the selected isolates

The six LAB isolates that produced the highest cholesterol removal activity were identified and the results were as the following:

#### 3.2.1. Morphological features

Microscopical examination after Gram stain revealed that all isolates were Gram-positive, four out of six isolates (isolate no.1, 16, 17, and 35) had a cocci-shape while the remaining two isolates (Isolate no. 33 and 34) had a bacilli-shape.

#### 3.2.2. Biochemical characterization

All isolates were catalase-negative. Carbohydrate assimilation and fermentation of 49 substances with one control were identified on API 50 CH strips and the results are shown in **S 8** (Supporting Information). Following the manufacturer protocol, referring to the analytical profile index and by using the APIWEB’S microbial identification systems software, the isolates were identified as presented in Table 1.

**Table 1:** Identification of the six LAB isolates based on the interpretation of the API 50 CH strips.

Isolate No.	Genus	Species
**17**	*Pediococcus*	N/A
**35**	*Pediococcus*	N/A
**33**	*Lactobacillus*	*acidophilus*
**34**	*Lactobacillus*	*acidophilus*
**1**	*Lactococcus*	*Lactis* ssp.* lactis*
**16**	*Lactococcus*	*lactis *ssp. *lactis*

N/A, data obtained were enough only for the identification of the genus level.

The six LAB isolates belonged to three genera *Lactobacillus*, *Lactococcus*, and *Pediococcus*. Both isolates no. 17 and 35 were identified only to the genus level as *Pediococcus*. Isolates no. 33 and 34 were identified as *Lactobacillus acidophilus* (*L*. *acidophilus*) while isolates no. 1 and 16 belonged to the *Lactococcus* genus and were identified as *lactis* ssp. *lactis*.

### 3.3. Animal feeding trial: Effect of two selected LAB species on serum lipid levels of diet-induced hyperlipidemic hamsters

After a 28 days animal feeding trial, serum TC, TG, LDL-C, and HDL-C levels were determined in the different tested animal groups.

#### 3.3.1 Effect of administration of two selected LAB isolates on serum TC levels

**Fig 3:**
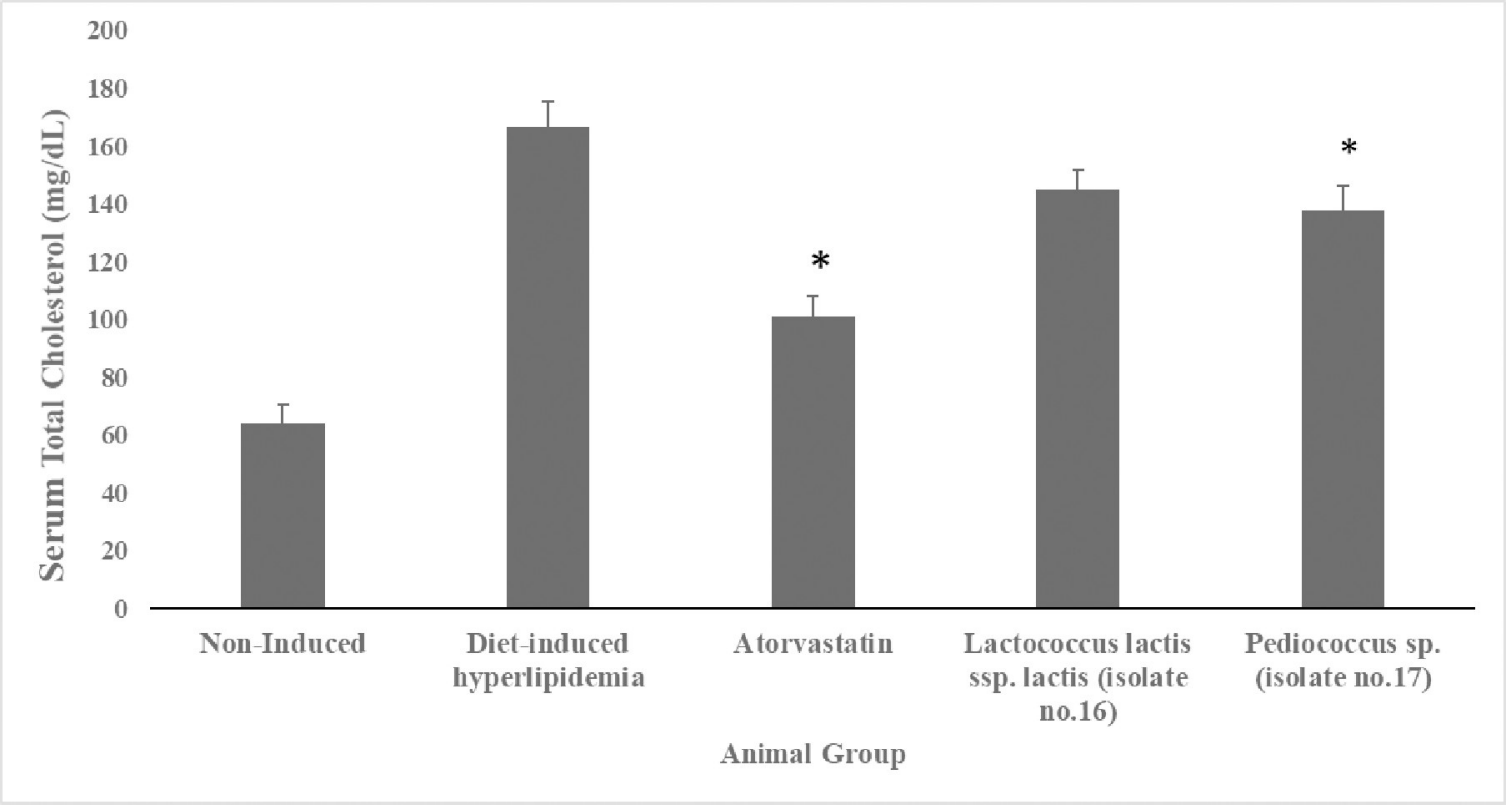
Serum total cholesterol levels (mg/dL) of hyperlipidemic hamsters. Non-induced group (standard food diet), diet-induced hyperlipidemic group (HFD-fed), *Lactococcus lactis* ssp. *lactis* group (HFD-fed + 1×10^9^ CFU/mL /day), *Pediococcus* sp. group (HFD-fed + 1×10^9^ CFU/mL /day) and atorvastatin group (HFD-fed + 1.23 mg/kg/day atorvastatin). Data are represented as the mean ±SEM (n = 6). Asterisks indicate a significant difference (*p*<0.05).

As shown in Fig 3 and S3 Table (Supporting Information), serum TC levels in the diet-induced hyperlipidemic group (166.5mg/dL) were significantly (*p* <0.05) higher compared to the non-induced group (64 mg/dL) by 61.6%. TC levels was significantly (*p* <0.05) lower by 39.2% and 17.3% in the atorvastatin (101.2mg/dL) and the *pediococcus* sp. (137. mg/dL) supplemented groups, respectively, in comparison to the diet-induced hyperlipidemic group (166.5mg/dL). Whereas, the TC levels of the *Lactococcus lactis* ssp. *lactis* supplemented group was 145.2mg/dL with no significant difference (*p* >0.05) as compared to the diet-induced hyperlipidemic group.

#### 3.3.2. Effect of administration of two selected LAB isolates on serum TG levels

**Fig 4:**
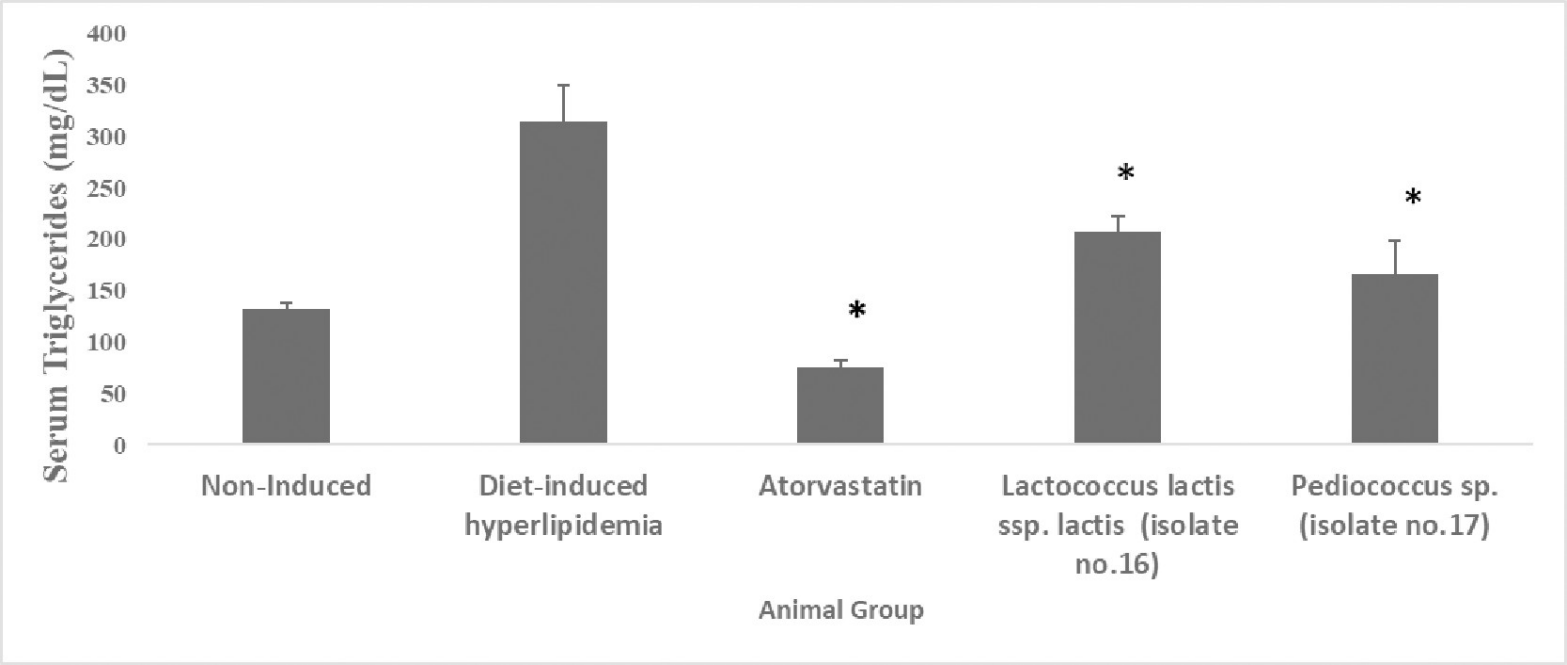
Serum triglycerides levels (mg/dL) of hyperlipidemic hamsters. Non-induced group (standard food diet), diet-induced hyperlipidemic group (HFD-fed), *Lactococcus lactis* ssp. *lactis* group (HFD-fed + 1×10^9^ CFU/mL /day), *Pediococcus* sp. group (HFD-fed + 1×10^9^ CFU/mL /day) and atorvastatin group (HFD-fed + 1.23 mg/kg/day atorvastatin). Data are represented as the mean ±SEM (n = 6). Asterisks indicate a significant difference (*p*<0.05).

The results in Fig 4 and S4 Table (Supporting Information) demonstrate that serum TG levels were significantly (*p* <0.05) elevated by 58.1% in the diet-induced hyperlipidemic group (315.2 mg/dL) compared to the non-induced group (132 mg/dL). TG levels were significantly (*p* <0.05) lower in all the supplemented groups, the decrease was 76.0%, 34.3% and 47.0% in atorvastatin (75.5mg/dL), *Lactococcus lactis* ssp. *lactis* (207.2 mg/dL), and *Pediococcus* sp. (167mg/dL) supplemented group, respectively, in contrast to the diet-induced hyperlipidemic group.

#### 3.3.3. Effect of administration of two selected LAB isolates on serum HDL-C levels

**Fig 5:**
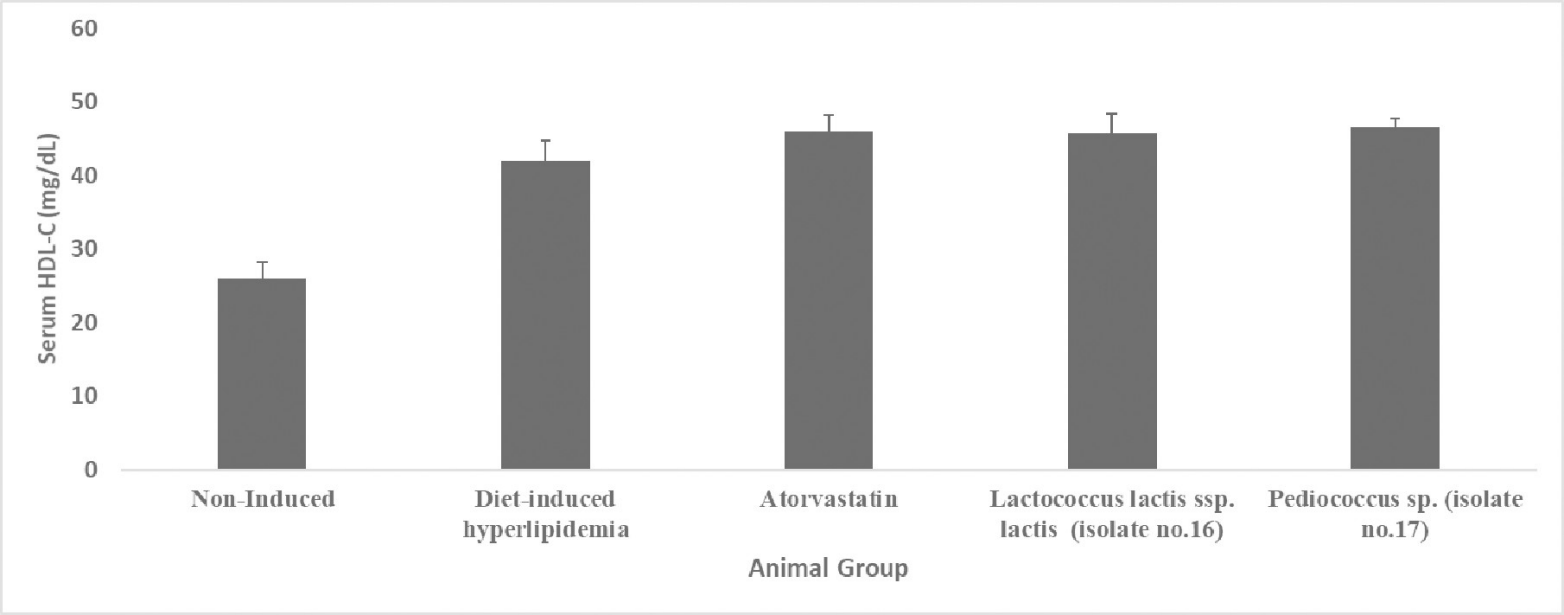
Serum HDL-C level (mg/dL) of hyperlipidemic hamsters. Non-induced group (standard food diet), diet-induced hyperlipidemic group (HFD-fed), *Lactococcus lactis* ssp. *lactis* group (HFD-fed + 1×10^9^ CFU/mL /day), *Pediococcus* sp. group (HFD-fed + 1×10^9^ CFU/mL /day) and atorvastatin group (HFD-fed + 1.23 mg/kg/day atorvastatin). Data are represented as the mean ±SEM (n = 6). Asterisks indicate a significant difference (*p*<0.05).

As represented in Fig 5 and S5 Table (Supporting Information), serum HDL-C levels in the diet-induced hyperlipidemic group (42 mg/dL) were significantly (*p*<0.05) higher by 38.1% compared to the non-induced group (26 mg/dL). Serum HDL-C levels were higher by 8.3%, 8.7%, and 9.7% in the *Lactococcus lactis* ssp. *lactis* (45.8 mg/dL), atorvastatin (46mg/dL), and *Pediococcus* sp. (46.5 mg/dl) supplemented groups, respectively, compared to the diet-induced hyperlipidemic group. However, this minimal rise was statistically non-significant (*p* >0.05) in all the three supplemented groups.

#### 3.3.4. Effect of administration of two selected LAB isolates on serum LDL-C levels

**Fig 6:**
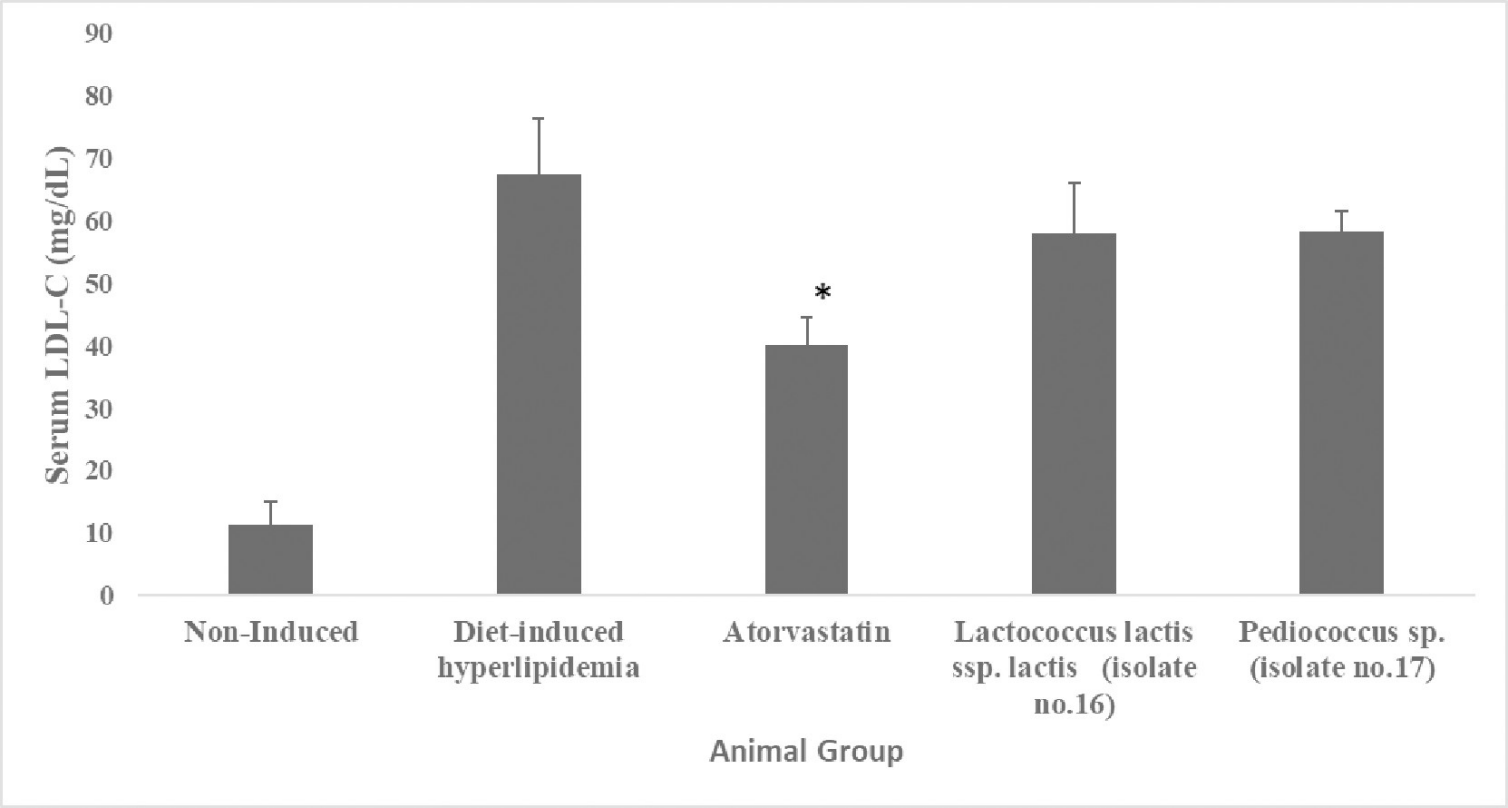
Serum LDL-C levels (mg/dL) of hyperlipidemic hamsters Non-induced group (standard food diet), diet-induced hyperlipidemic group (HFD-fed), *Lactococcus lactis* ssp. *lactis* group (HFD-fed + 1×10^9^ CFU/mL /day), *Pediococcus* sp. group (HFD-fed + 1×10^9^ CFU/mL /day) and atorvastatin group (HFD-fed + 1.23 mg/kg/day atorvastatin). Data are represented as the mean ±SEM (n = 6). Asterisks indicate a significant difference (*p*<0.05).

Additionally, the levels of LDL-C were measured among the animal groups (data are illustrated in Fig 6 and S6 Table (Supporting Information)). Serum LDL-C levels in the diet-induced hyperlipidemic group (67.3 mg/dL) were significantly (*p* <0.05) higher by 82.9% in contrast to the non-induced group (11.5 mg/dL). LDL-C levels were significantly (*p* <0.05) lower by 40.3% in the atorvastatin-supplemented group (40.2 mg/dL) compared to the diet-induced hyperlipidemic group. However, LDL-C levels were none significantly decreased (*p* >0.05) in both *Lactococcus lactis* ssp. *lactis* (58mg/dL) and *Pediococcus* sp. supplemented groups (58.2mg/dl) when compared to the diet-induced hyperlipidemic group.

## 4. Discussion

As hyperlipidemia causes an array of disorders [49], substantial effort has been expended in developing various medications against it. However, those lipid-lowering medications agents have been reported to cause various adverse effects [7], which is why probiotics with their natural and safe characteristics offer a promising approach.

Exploring new *Lactococcal* probiotic strains would be favorable since they can grow well in milk, apart from *L*. *acidophilus* strains during the production of fermented milk products [50]. According to [51], developing or selecting a new probiotic necessitates a previous assessment of its safety, and *Lactococcus* sp. have a considerable advantage in this regard because they are frequently given the GRAS status, as are *Lactobacillus* species[50].

*Lactobacillus* and *Bifidobacterium* species are two examples of probiotics that have been well studied for their hypolipidemic effects in both human and animal studies [52]. Still, there is a gap between the number of discovered strains and the different needs of humans and industry [35]. That is why we ought to discover more bacterial strains and that is the target of our research, to discover other novel bacteria with potential anti-hypercholesterolemic activity.

### 4.1. Removal of cholesterol by the recovered LAB isolates

In this study, we isolated 50 LAB isolates and screened them for their *in vitro* cholesterol removal ability. Then we selected the six isolates that produced the highest cholesterol removal percentages to be identified and used in the rest of the study.

### 4.2. Characterization and identification of the selected isolates

Isolation of LAB is used to detect and ascertain the genus of the bacterial isolates. Isolation was carried out on MRS agar because it is a selective medium and considered as the most common medium used to grow LAB, including *Pediococcus*, *Lactobacillus*, *Streptococcus*, and *Leuconostoc* bacterial genera according to [53]. Macroscopic observation and microscopic examination were carried out after culturing on an MRS agar plate. Macroscopic observations of the isolated colonies revealed a circular shape with an entire margin, convex elevation, and milky white color. Such characters of LAB that we isolated are similar to what’s described by [54–56]. As for the microscopic examination and catalase test, all the six isolates were catalase-negative, Gram-positive with isolates no. 1, 16, 17, and 35 a cocci-shape while isolates no. 33 and 34 having a bacilli-shape, such findings come in agreement with [57].

The carbohydrate fermentation pattern of the isolates was tested using the API 50 CH and analyzed by the APIWEB. Based on systematic databases, the API 50 CH is a cost-effective, easily operated, and well-recognized technique for the identification of microorganisms. The platform features a huge and reliable database that is obtainable through the internet-based APIWEB [58].

After identifying the isolates, we could state that according to the results in Fig 2, *pediococcus* sp. (isolates no. 17 and 35) had good cholesterol removal abilities of 36.5% ±7.442 and 40.5% ±13.208, respectively. Similar cholesterol removal ability was shown by the *L*. *acidophilus* species (isolates no.33 and 34) with the values of 40.0% ±15.88 and, 39.0% ±6.604 respectively. Lastly, *Lactococcus lactis* ssp. *lactis* (isolates no.1 and 16) showed the highest cholesterol removal rates of (55.6% ±10.166 and 47.1% ±7.205), respectively.

### 4.3 Animal feeding trial

*Lactococcus/Pediococcus* are conventionally not thought to be natural inhabitants of the human gastrointestinal tract which is why limited studies exist on their probiotic activity, while *Lactobacillus* genera are extensively studied and considered as the most widely recognized probiotic [32,52,59]. That prompted us to use those less studied genera for the *in vivo* cholesterol removal activity study in order to produce better insights into them. Out of each of these two selected genera, the species that had the most reproducible result in the in *vitro* cholesterol removal screening (Fig 2) were selected for the animal feeding trial.

#### 4.3.1 Effect of two selected LAB isolates on serum lipid levels of hyperlipidemic hamsters

For the animal study, we have gone through two very important questions: the first one was which animal is better to be used? We found out that the golden Syrian hamsters have been increasingly used in the lipoprotein metabolism research, and the investigation of the hypolipidemic agents’ effect, like PPAR agonists, statins, and CETP inhibitors [60]. The reason for that is that these hamsters resemble humans in terms of lipid utilization and high susceptibility to dietary cholesterol [61]. Upon isolation and characterization of the hamster LDL receptor gene [62], it showed a high similarity to that of humans. The synthesis of bile acids and hepatic cholesterol is also similar in both hamsters and humans, which makes them prone to hypercholesterolemia induced by an increased dietary cholesterol diet, on the other hand, rats are resistant to this[63]. For all the above-mentioned reasons, Syrian hamsters have already been used to observe many pharmacological agents that have different modes of action [64]. Moreover, hamsters are considered to be a better model than rats in the study of the potential probiotics cholesterol-lowering effect because hamsters show are similar to humans in bile acid composition and synthesis [65].

After we settled on the choice of the golden Syrian hamster as the animal model to be used in our study, we needed to answer the second question; when to supplement the LAB isolates or the standard drug? To get the answer we have gone through the literature and we found that the lipid-lowering effect could be measured using different study designs, one of them induces hyperlipidemia first then after a while introduces the LAB isolates [66,67]. The other approach induces hyperlipidemia simultaneously with the supplementation of LAB isolates. This latter approach was used in many researches like the screening for cholesterol-lowering probiotics from LAB isolated from corn silage [38], the investigation of the hypocholesterolemic activity of *Lactobacillus casei*-fermented milk in hamsters [68], the comparison between hamsters and rats as models for evaluation of the hypocholesterolemic efficacy of potential probiotics [65], the detection of the lipid-lowering effects of *Pediococcus acidilactici* in high fat diet-induced mice [69], investigation of the hypocholesterolemic effect of two *Lactococcus lactis* subspecies [70] and the detection of the effect of *Pediococcus pentosaceus* PP04 on high-fat diet-induced hyperlipidemia[71]. We chose to use that design in our work.

*4*.*3*.*1*.*1 Effect on serum TC levels*. As shown in Fig 3, *Pediococcus* sp. successfully reduced serum TC levels *(p <0*.*05)* by 17.3% (137.7 mg/dL) 4 weeks post-administration in comparison to the diet-induced hyperlipidemic group. Our results come in agreement with [66,69]. Several hypotheses were put to explain the mechanism by which probiotics could produce this hypocholesterolemic effect: by the intestinal bacteria which consume cholesterol decreasing its available amount for absorption [24]. Cholesterol can bind to the bacterial cell surface [25], be implemented into bacterial membranes [26], or be transformed into coprostanol by the cholesterol reductase enzyme that is produced by *Lactobacilli* strains [27]. Another proposed mechanism is through suppression of micelle formation by a specific probiotic strain [72]. Also, short-chain fatty acids produced by gut microflora during selective fermentation of food may reduce blood cholesterol levels [73]. Lastly, some bacterial species secrete bile salt hydrolase, resulting in enhanced bile excretion in the stool [74].

As for *Lactococcus lactis* ssp. *Lactis* supplemented group, resulted in a modest decrease in the serum TC levels by 12.8%, however, this reduction was considered to be non-statistically significant (*p = 0*.*05*), although it demonstrated a good cholesterol removal ability *in vitro*.

The lack of the *in vivo* effect despite having an *in vitro* one could be due to the fact that inside the body in the duodenum and upper jejunum where cholesterol absorption mainly occurs [75] have high bile salt concentrations [76] that dramatically inhibit the growth of bacteria and consequently inhibit their *in vivo* effect [38]. A study reported a contradicted result where supplementation of *Lactococcus lactis* ssp. *lactis* 527 (LACC-527) strain significantly decreased serum cholesterol levels in the Sprague-Dawley rats after 6 weeks post-administration [70]. This disagreement with our finding could be due to the difference in the used bacterial strain, for instance, the tolerance to bile salts has been shown to vary among *Lactococcus* strains [50], also could be due to differences in the animal model or the period of the experiment [70].

*4*.*3*.*1*.*2*. *Effect on serum TG levels*. The results in Fig 4 showed that both *Pediococcus* sp. and *Lactococcus lactis* ssp. *lactis* supplementation successfully reduced the serum TG levels (*p <0*.*05*) by 47.0% (167 mg/dL) and 34.3% (207.2 mg/dL), respectively, 4 weeks post-administration as compared to the diet-induced hyperlipidemic group. In addition, these findings agree with [71] which reported a significant reduction in the serum TG levels following 8 weeks feeding period of *Pediococcus pentosaceus* in diet-induced hyperlipidemic mice. However, our results disagree with [69] which reported no significant difference in the serum TG levels between groups after supplementation of *Pediococcus acidilactici* M76 in high-fat diet-induced obese mice. Also, as for *Lactococcus lactis* ssp. *lactis* effect on TG level, similar results were reported by [70].

Figs 3 and 4 show that atorvastatin, a standard drug used in the market for the treatment of hypercholesterolemia had a higher reduction effect on the serum TC and TG levels than the two tested LAB isolates. The *Pediococcus* sp. supplemented group resulted in a 17.3% decrease in the TC level of the HFD-fed hamsters, in comparison to atorvastatin with a 39.2% decrease. On the other hand, the effect on serum TG levels was more pronounced; *the Pediococcus* sp. supplemented group demonstrated a 47.0% decrease while atorvastatin resulted in a 76.0% decrease. Still, given the reported side effects by atorvastatin [7], such differences in the hypolipidemic effect could be in favor of the use of probiotics as an adjuvant therapy to decrease the atorvastatin dose and consequently its side effects, which will ultimately improve the patient compliance.

*4*.*3*.*1*.*3*. *Effect on serum HDL and LDL cholesterol levels*. Neither *Pediococcus* sp. (isolate no.17) nor *Lactococcus lactis* ssp. *lactis* (isolate no.16) supplementation was able to significantly (*P > 0*.*05*) affect the serum HDL-C or LDL-C levels (Figs 5 and 6). Our results come in agreement with [70] who reported no significant difference in HDL-cholesterol levels between the control and *Lactococcus lactis* ssp. *lactis* 527 (LACC-527) supplemented groups. In addition, [71] reported that the supplementation of *Pediococcus strain PP04* for 8 weeks had no effect on the serum HDL-C levels in mice.

Lactococcus lactis ssp. lactis 527 demonstrated similar results to our findings of no significant effect on serum LDL-C levels [70]. However, contradictory results were reported by [66] that demonstrated a significant reduction in the serum LDL-C levels caused by *Pediococcus pentosaceus* strain, KID7. Furthermore, another study reported an LDL-C reduction effect caused by *Pediococcus pentococcus* PP04 [71].

## 5. Conclusion

In this study, a total of 50 LAB were isolated from raw milk (human and animal) and dairy products. Preliminary screening was carried out to assess the cholesterol removal capabilities of the recovered LAB isolates *in vitro*. The six LAB isolates showing the highest cholesterol removal capabilities were morphologically and biochemically characterized. Finally, two bacterial candidates (*Pediococcus and Lactococcus*) were selected for evaluation of their potential hypolipidemic activity using a laboratory animal model. Both *Pediococcus* sp. and *Lactococcus lactis* ssp. *lactis* significantly (*p* <0.05) reduced serum TG levels by 47.0% and 34.3%, respectively, four weeks post-administration. Conversely, only *Pediococcus* sp. was able to significantly (*p <0*.*05*) reduce serum TC levels by 17.3%. However, neither *Pediococcus* sp. nor *Lactococcus lactis* ssp. *lactis* was able to significantly affect serum HDL-C or LDL-C levels. Further investigations are needed to understand the mechanism by which these bacterial isolates exert their hypolipidemic activity. In addition, extensive research is urgently recommended for the production of probiotic-based therapeutic agents against hyperlipidemia.

## Supporting information

S1 Table Preliminary screening dataset. (XLSX)

S2 Table GC replicates dataset. (XLSX)

S3 Table Serum cholesterol dataset. (XLSX)

S4 Table Serum triglycerides dataset. (XLSX)

S5 Table Serum HDL-C dataset. (XLSX)

S6 Table Serum LDL-C dataset. (XLSX)

S7 Table Viable count dataset. (XLSX)

S1 File (PDF)

## References

[1] Abbafati C, Abbas KM, Abbasi-Kangevari M, Abd-Allah F, Abdelalim A, Abdollahi M, et al. Global burden of 369 diseases and injuries in 204 countries and territories, 1990–2019: a systematic analysis for the Global Burden of Disease Study 2019. The Lancet [Internet]. 2020 Oct 17 [cited 2021 Nov 29];396(10258):1204–22. Available from: http://www.thelancet.com/article/S0140673620309259/fulltext. doi: 10.1016/S0140-6736(20)30925-9 33069326 PMC7567026

[2] Mozaffarian D, Benjamin EJ, Go AS, Arnett DK, Blaha MJ, Cushman M, et al. Heart Disease and Stroke Statistics—2016 Update. Circulation [Internet]. 2016 Jan 26 [cited 2021 Nov 29];133(4):e38–48. Available from: https://www.ahajournals.org/doi/abs/10.1161/cir.0000000000000350. 26673558 10.1161/CIR.0000000000000350

[3] Walrand S. Nutrition and skeletal muscle. Nutrition and Skeletal Muscle. 2018 Jan 1;1–574.

[4] Piggott JR, Conner JM. Whisky, Whiskey, and Bourbon. Encyclopedia of Food Sciences and Nutrition. 2003;6171–7.

[5] Cleeman JI. Executive Summary of The Third Report of The National Cholesterol Education Program (NCEP) Expert Panel on Detection, Evaluation, And Treatment of High Blood Cholesterol In Adults (Adult Treatment Panel III). JAMA [Internet]. 2001 May 16 [cited 2021 Nov 29];285(19):2486–97. Available from: https://pubmed.ncbi.nlm.nih.gov/11368702/. doi: 10.1001/jama.285.19.2486 11368702

[6] C E-K T G, S B, T M. Inflammatory markers and cardiovascular risk in the metabolic syndrome. Frontiers in bioscience (Landmark edition) [Internet]. 2011 Jan 1 [cited 2021 Sep 15];16(5):1663–74. Available from: https://pubmed.ncbi.nlm.nih.gov/21196255/. 10.2741/3812 21196255

[7] Zodda D, Giammona R, Schifilliti S. Treatment Strategy for Dyslipidemia in Cardiovascular Disease Prevention: Focus on Old and New Drugs. Pharmacy. 2018;6(1):10. doi: 10.3390/pharmacy6010010 29361723 PMC5874549

[8] Feingold KR. Cholesterol Lowering Drugs. Feingold KR, Anawalt B, Boyce A, Chrousos G, Dungan K, Grossman A, et al., editors. NCBI Bookshelf [Internet]. 2021 Mar 30 [cited 2021 Sep 26]; Available from: https://www.ncbi.nlm.nih.gov/books/NBK395573/.

[9] GV M. Studies of a surfactant and cholesteremia in the Maasai. The American journal of clinical nutrition [Internet]. 1974 [cited 2021 Sep 26];27(5):464–9. Available from: https://pubmed.ncbi.nlm.nih.gov/4596028/. 10.1093/ajcn/27.5.464 4596028

[10] Nazir Y, Hussain SA, Hamid AA, Song Y. Probiotics and Their Potential Preventive and Therapeutic Role for Cancer, High Serum Cholesterol, and Allergic and HIV Diseases. BioMed Research International [Internet]. 2018 [cited 2021 Sep 27];2018. Available from: https://www.readcube.com/articles/10.1155%2F2018%2F3428437. 10.1155/2018/3428437 PMC6136537 30246019

[11] WH H, P H, R G, J B, U S. Taxonomy and important features of probiotic microorganisms in food and nutrition. The American journal of clinical nutrition [Internet]. 2001 [cited 2021 Sep 15];73(2 Suppl). Available from: https://pubmed.ncbi.nlm.nih.gov/11157343/. doi: 10.1093/ajcn/73.2.365s 11157343

[12] Harzallah D, Belhadj H. Lactic Acid Bacteria as Probiotics: Characteristics, Selection Criteria and Role in Immunomodulation of Human GI Muccosal Barrier. Lactic Acid Bacteria—R & D for Food, Health and Livestock Purposes [Internet]. 2013 Jan 30 [cited 2021 Sep 15]; Available from: https://www.intechopen.com/chapters/42329.

[13] Fujisawa T, Benno Y, Yaeshima T, Mitsuoka T. Taxonomic Study of the Lactobacillus acidophilus Group, with Recognition of Lactobacillus gallinarum sp. nov. and Lactobacillus johnsonii sp. nov. and Synonymy of Lactobacillus acidophilus Group A3 (Johnson et al. 1980) with the Type Strain of Lactobacill. International Journal of Systematic and Evolutionary Microbiology [Internet]. 1992 Jul 1 [cited 2021 Sep 15];42(3):487–91. Available from: https://www.microbiologyresearch.org/content/journal/ijsem/10.1099/00207713-42-3-487. 10.1099/00207713-42-3-487 1503977

[14] K G, AE S, TM H, PL R, CL F, WE S. Inverse association of H2O2-producing lactobacilli and vaginal Escherichia coli colonization in women with recurrent urinary tract infections. The Journal of Infectious Diseases [Internet]. 1998 Aug 1 [cited 2021 Sep 15];178(2):446–50. Available from: https://europepmc.org/article/med/9697725. doi: 10.1086/515635 9697725

[15] Vlková E, Medková J, Rada V. Comparison of four methods for identification of bifidobacteria to the genus level. Czech Journal of Food Sciences. 2018 Feb 11;20(No. 5):171–4.

[16] G K, A P, C B, G R. Taxonomy and physiology of probiotic lactic acid bacteria. International journal of food microbiology [Internet]. 1998 May 26 [cited 2021 Sep 15];41(2):103–25. Available from: https://pubmed.ncbi.nlm.nih.gov/9704860/. doi: 10.1016/s0168-1605(98)00049-x 9704860

[17] M F, M N. Effects of a mixture of organisms, Lactobacillus acidophilus or Streptococcus faecalis on cholesterol metabolism in rats fed on a fat- and cholesterol-enriched diet. The British journal of nutrition. 1996 Dec;76(6):857–67. doi: 10.1079/bjn19960092 9014654

[18] SE G, CR N, C M. Assimilation of cholesterol by Lactobacillus acidophilus. Applied and environmental microbiology [Internet]. 1985 [cited 2021 Sep 15];49(2):377–81. Available from: https://pubmed.ncbi.nlm.nih.gov/3920964/. doi: 10.1128/aem.49.2.377-381.1985 3920964 PMC238411

[19] Nguyen TDT, Kang JH, Lee MS. Characterization of Lactobacillus plantarum PH04, a potential probiotic bacterium with cholesterol-lowering effects. International Journal of Food Microbiology. 2007 Feb 15;113(3):358–61. doi: 10.1016/j.ijfoodmicro.2006.08.015 17140690

[20] Agerbaek M, Gerdes LU, Richelsen B. Hypocholesterolaemic effect of a new fermented milk product in healthy middle-aged men. European Journal of Clinical Nutrition. 1995;49(5):346–52. 7664720

[21] Anderson JW, Gilliland SE. Effect of Fermented Milk (Yogurt) Containing Lactobacillus Acidophilus L1 on Serum Cholesterol in Hypercholesterolemic Humans. Journal of the American College of Nutrition. 1999 Feb 1;18(1):43–50. doi: 10.1080/07315724.1999.10718826 10067658

[22] Keim NL, Marlett JA, Amundson CH. The cholesterolemic effect of skim milk in young men consuming controlled diets. Nutrition Research. 1981;1(5):429–42.

[23] JZ X, S K, N T, K M, K O, A H, et al. Effects of milk products fermented by Bifidobacterium longum on blood lipids in rats and healthy adult male volunteers. Journal of dairy science [Internet]. 2003 [cited 2021 Sep 15];86(7):2452–61. Available from: https://pubmed.ncbi.nlm.nih.gov/12906063/. doi: 10.3168/jds.S0022-0302(03)73839-9 12906063

[24] DI P, GR G. Cholesterol assimilation by lactic acid bacteria and bifidobacteria isolated from the human gut. Applied and environmental microbiology [Internet]. 2002 Sep [cited 2021 Sep 15];68(9):4689–93. Available from: https://pubmed.ncbi.nlm.nih.gov/12200334/. doi: 10.1128/AEM.68.9.4689-4693.2002 12200334 PMC124114

[25] MT L, NP S. Acid and bile tolerance and cholesterol removal ability of lactobacilli strains. Journal of dairy science [Internet]. 2005 [cited 2021 Sep 15];88(1):55–66. Available from: https://pubmed.ncbi.nlm.nih.gov/15591367/. doi: 10.3168/jds.S0022-0302(05)72662-X 15591367

[26] Lye HS, Rahmat-Ali GR, Liong MT. Mechanisms of cholesterol removal by lactobacilli under conditions that mimic the human gastrointestinal tract. International Dairy Journal. 2010 Mar 1;20(3):169–75.

[27] HS L, G R, MT L. Removal of cholesterol by lactobacilli via incorporation and conversion to coprostanol. Journal of dairy science [Internet]. 2010 Apr [cited 2021 Sep 15];93(4):1383–92. Available from: https://pubmed.ncbi.nlm.nih.gov/20338415/. doi: 10.3168/jds.2009-2574 20338415

[28] Hatakka K, Saxelin M, Mutanen M, Korpela R, Hatakka K, Holma R, et al. Lactobacillus rhamnosus LC705 Together with Propionibacterium freudenreichii ssp shermanii JS Administered in Capsules Is Ineffective in Lowering Serum Lipids. Journal of the American College of Nutrition. 2008 Aug 1;27(4):441–7. doi: 10.1080/07315724.2008.10719723 18978162

[29] Simons LA, Amansec SG, Conway P. Effect of Lactobacillus fermentum on serum lipids in subjects with elevated serum cholesterol. Nutrition, Metabolism and Cardiovascular Diseases. 2006 Dec;16(8):531–5. doi: 10.1016/j.numecd.2005.10.009 17126768

[30] Xie N, Cui Y, Yin YN, Zhao X, Yang JW, Wang ZG, et al. Effects of two Lactobacillus strains on lipid metabolism and intestinal microflora in rats fed a high-cholesterol diet. BMC Complementary and Alternative Medicine. 2011;11. 21722398 10.1186/1472-6882-11-53 PMC3144010

[31] Merenstein DJ, Sanders ME, Tancredi DJ. Probiotics as a Tx resource in primary care. The Journal of family practice. 2020 Apr 1;69(3):E1–10. 32289131

[32] Jiang S, Cai L, Lv L, Li L. Pediococcus pentosaceus, a future additive or probiotic candidate. Microbial Cell Factories [Internet]. 2021;20(1):1–14. Available from: doi: 10.1186/s12934-021-01537-y PMC7885583 33593360

[33] Degnan King FH, Washington S, Degnan FH. The US Food and Drug Administration and Probiotics: Regulatory Categorization. Clinical Infectious Diseases [Internet]. 2008 Feb 1 [cited 2021 Nov 10];46(Supplement_2):S133–6. Available from: https://academic.oup.com/cid/article/46/Supplement_2/S133/277296. doi: 10.1086/523324 18181719

[34] Koutsoumanis K, Allende A, Alvarez-Ordóñez A, Bolton D, Bover-Cid S, Chemaly M, et al. Update of the list of QPS-recommended biological agents intentionally added to food or feed as notified to EFSA 13: suitability of taxonomic units notified to EFSA until September 2020. EFSA Journal. 2021 Jan 1;19(1). 10.2903/j.efsa.2021.6377 PMC7842631 33537066

[35] Min BE, Hwang HG, Lim HG, Jung GY. Optimization of industrial microorganisms: recent advances in synthetic dynamic regulators. Vol. 44, Journal of Industrial Microbiology and Biotechnology. Springer Verlag; 2017. p. 89–98. 27832388 10.1007/s10295-016-1867-y

[36] Sallam MK, Wali IE, Attia AEFMH. Isolation of Lactobacilli and Bifidobacteria Species from Human Breast Milk. The Egyptian Journal of Medical Microbiology. 2015;24(3):69–73.

[37] Reuben RC, Roy PC, Sarkar SL, Alam RU, Jahid IK. Isolation, characterization, and assessment of lactic acid bacteria toward their selection as poultry probiotics. BMC Microbiology. 2019;19(1):1–20. 31718570 10.1186/s12866-019-1626-0 PMC6852909

[38] Ma C, Zhang S, Lu J, Zhang C, Pang X, Lv J. Screening for cholesterol-lowering probiotics from lactic acid bacteria isolated from corn silage based on three hypothesized pathways. International Journal of Molecular Sciences. 2019 May 1;20(9). doi: 10.3390/ijms20092073 31035460 PMC6539855

[39] Akram AA, Abdel-Hamied MR, Magdy MO, Salha GD, Nehal K. Cholesterol reduction in vitro by novel probiotic lactic acid bacterial strains of Enterococcus isolated from healthy infants stool. African Journal of Microbiology Research. 2017;11(38):1434–44.

[40] Fletouris DJ, Botsoglou NA, Psomas IE, Mantis AI. Rapid Determination of Cholesterol in Milk and Milk Products by Direct Saponification and Capillary Gas Chromatography. Journal of Dairy Science. 1998;81(11):2833–40. doi: 10.3168/jds.S0022-0302(98)75842-4 9839224

[41] Madigan Michael T., Martinko John M., Stahl David A., Clark David P. Brock Biology of Microorganisms: Industrial & Scientific. 13th Edition. 2012.

[42] Harrigan Wilkie. Laboratory methods in Microbiology 3rd Edition. Academics Press, Califonia, USA. 1998.

[43] Ru X, Zhang CC, Yuan YH, Yue TL, Guo CF. Bile salt hydrolase activity is present in nonintestinal lactic acid bacteria at an intermediate level. Applied Microbiology and Biotechnology. 2019;103(2):893–902. doi: 10.1007/s00253-018-9492-5 30421106

[44] Campbell J. Optical Density Measurement. Definitions. 2020;3(3):1–20.

[45] WC H, YM C, NW K, CS H, L W, CH C, et al. Hypolipidemic effects and safety of Lactobacillus reuteri 263 in a hamster model of hyperlipidemia. Nutrients. 2015 May 15;7(5):3767–82. doi: 10.3390/nu7053767 25988768 PMC4446778

[46] Chen WC, Huang WC, Chiu CC, Chang YK, Huang CC. Whey protein improves exercise performance and biochemical profiles in trained mice. Medicine and Science in Sports and Exercise. 2014;46(8):1517–24. doi: 10.1249/MSS.0000000000000272 24504433 PMC4186725

[47] Animals NRC (US) C for the U of the G for the C and U of L. Guide for the Care and Use of Laboratory Animals. Guide for the Care and Use of Laboratory Animals [Internet]. 2011 Dec 27 [cited 2021 Sep 14]; Available from: https://www.ncbi.nlm.nih.gov/books/NBK54050/.

[48] Paigen B, Morrow A, Brandon C, Mitchell D, Holmes P. Variation in susceptibility to atherosclerosis among inbred strains of mice. Atherosclerosis [Internet]. 1985 Oct 1 [cited 2021 Sep 14];57(1):65–73. Available from: http://www.atherosclerosis-journal.com/article/0021915085901388/fulltext. 10.1016/0021-9150(85)90138-8 3841001

[49] S G, U L. The Year in Cardiology 2013: cardiovascular disease prevention. European heart journal [Internet]. 2014 Feb 1 [cited 2021 Oct 2];35(5):307–12. Available from: https://pubmed.ncbi.nlm.nih.gov/24385374/. doi: 10.1093/eurheartj/eht551 24385374

[50] Kimoto H, Kurisaki J, Tsuji NM, Ohmomo S, Okamoto T. Lactococci as probiotic strains: adhesion to human enterocyte-like Caco-2 cells and tolerance to low pH and bile. Vol. 29, Letters in Applied Microbiology. 1999. doi: 10.1046/j.1365-2672.1999.00627.x 10664972

[51] Salminen S, von Wright A, Morelli L, Marteau P, Brassart D, de Vos WM, et al. Demonstration of safety of probiotics—A review. International Journal of Food Microbiology. 1998;44(1–2). 10.1016/s0168-1605(98)00128-7 9849787

[52] Kimoto-Nira H, Mizumachi K, Nomura M, Kobayashi M, Fujita Y, Okamoto T, et al. Lactococcus sp. as potential probiotic lactic acid bacteria. Japan Agricultural Research Quarterly. 2007;41(3):181–9.

[53] Zahoor T, Rahman SU, Farooq U. Viability of Lactobacillus bulgaricus as Yoghurt Culture Under Different Preservation Methods. International Journal of Agriculture & Biology [Internet]. 2003;(September 2014):46–8. Available from: http://www.ijab.org.

[54] Sameen A, Anjum FM, Huma N, Khan MI. Comparison of locally isolated culture from Yoghurt (Dahi) with commercial culture for the production of mozzarella cheese. International Journal of Agriculture and Biology. 2010;12(2):231–6.

[55] Sulmiyati, Said NS, Fahrodi DU, Malaka R, Maruddin F. The characteristics of lactic acid bacteria isolated from Indonesian commercial kefir grain. Malaysian Journal of Microbiology. 2018;14(7):632–9.

[56] Nur F, Hatta M, Natzir R, Djide MN. Isolation of Lactic Acid Bacteria as a Potential Probiotic in Dangke, a Traditional Food from Enrekang, Indonesia. International Journal of Sciences: Basic and Applied Research (IJSBAR) [Internet]. 2017;35(1):19–27. Available from: http://gssrr.org/index.php?journal=JournalOfBasicAndApplied.

[57] Axelsson L. (2004) Lactic Acid Bacteria Classification and Physiology. In Salminen S., Wright A.V. and Ouwehand A., Eds., Lactic Acid Bacteria Microbiological and Functional Aspects, 3rd Edition, Marcel Dekker, New York, 1–67.—References—Scientific Research Publishing [Internet]. [cited 2021 Oct 2]. Available from: https://www.scirp.org/(S(351jmbntvnsjt1aadkposzje))/reference/ReferencesPapers.aspx?ReferenceID=1993823.

[58] Simatende P, Siwela M, Gadaga TH. Identification of lactic acid bacteria and determination of selected biochemical properties in emasi and emahewu. South African Journal of Science. 2019;115(11–12).

[59] Sivamaruthi BS, Bharathi M, Kesika P, Suganthy N, Chaiyasut C. The Administration of Probiotics against Hypercholesterolemia: A Systematic Review. Applied Sciences 2021, Vol 11, Page 6913 [Internet]. 2021 Jul 27 [cited 2021 Nov 22];11(15):6913. Available from: https://www.mdpi.com/2076-3417/11/15/6913/htm.

[60] Kan CFK, Singh AB, Dong B, Shende VR, Liu J. PPARδ activation induces hepatic long-chain acyl-CoA synthetase 4 expression in vivo and in vitro. Biochimica et Biophysica Acta—Molecular and Cell Biology of Lipids. 2015;1851(5):577–87. doi: 10.1016/j.bbalip.2015.01.008 25645621 PMC5292870

[61] Moghadasian MH. Experimental atherosclerosis: A historical overview. Life Sciences. 2002 Jan 11;70(8):855–65. doi: 10.1016/s0024-3205(01)01479-5 11853223

[62] Bishop’ RW. Structure of the hamster low density lipoprotein receptor gene. Journal of Lipid Research. 1992;33. 1527478

[63] Chen W, Fan S, Xie X, Xue N, Jin X, Wang L. Novel PPAR Pan Agonist, ZBH Ameliorates Hyperlipidemia and Insulin Resistance in High Fat Diet Induced Hyperlipidemic Hamster. PLOS ONE [Internet]. 2014 Apr 23 [cited 2022 Mar 8];9(4):e96056. Available from: https://journals.plos.org/plosone/article?id=10.1371/journal.pone.0096056. 24759758 10.1371/journal.pone.0096056 PMC3997506

[64] Sima A, Stancu C, Constantinescu E, Ologeanu L, Simionescu M. The hyperlipemic hamster—A model for testing the anti-atherogenic effect of amlodipine. Journal of Cellular and Molecular Medicine. 2001;5(2):153–62. 12067498 10.1111/j.1582-4934.2001.tb00148.x PMC6738129

[65] Mnf-PW, Guo C-F, Yuan Y-H, Yue T-L, Li J-Y. FOOD & FUNCTION Hamsters Are a Better Model System than Rats for Evaluating the Hypocholesterolemic Efficacy of Potential Probiotic Strains. Available from: 10.1002/mnfr.201800170. 29939474

[66] K D, YS L, SA P, SH Y, JW S. Preliminary probiotic and technological characterization of Pediococcus pentosaceus strain KID7 and in vivo assessment of its cholesterol-lowering activity. Frontiers in microbiology [Internet]. 2015 [cited 2021 Oct 2];6(AUG). Available from: https://pubmed.ncbi.nlm.nih.gov/26300852/. doi: 10.3389/fmicb.2015.00768 26300852 PMC4523826

[67] Zhao MJ, Wang SS, Jiang Y, Wang Y, Shen H, Xu P, et al. Hypolipidemic effect of XH601 on hamsters of Hyperlipidemia and its potential mechanism. Lipids in Health and Disease. 2017 May 2;16(1). doi: 10.1186/s12944-017-0472-z 28464894 PMC5414347

[68] Guo C-F, Zhang S, Yuan Y-H, Li J-Y, Yue T-L. Probiotics www.mnf-journal.com Bile Salt Hydrolase and S-Layer Protein are the Key Factors Affecting the Hypocholesterolemic Activity of Lactobacillus casei-Fermented Milk in Hamsters. Available from: 10.1002/mnfr.201800728. 30346664

[69] Moon YJ, Baik SH, Cha YS. Lipid-lowering effects of pediococcus acidilactici M76 Isolated from Korean traditional makgeolli in high fat diet-induced obese mice. Nutrients. 2014 Mar 7;6(3):1016–28. doi: 10.3390/nu6031016 24609135 PMC3967175

[70] Lee W-K, Lim H-J, Kim S-Y, Kimoto H, Ohmomo S, Tashiro Y, et al. Hypocholesterolemic Effect of *Lactococcus lactis* subsp. *lactis* biovar *diacetylactis* N7 and *Lactococcus lactis* subsp. *lactis* 527 Strains in SD Rats. Bioscience and Microflora. 2005;24(1):11–6.

[71] Wang Y, You Y, Tian Y, Sun H, Li X, Wang X, et al. Pediococcus pentosaceus PP04 Ameliorates High-Fat Diet-Induced Hyperlipidemia by Regulating Lipid Metabolism in C57BL/6N Mice. Journal of Agricultural and Food Chemistry. 2020;68(51):15154–63. doi: 10.1021/acs.jafc.0c05060 33300795

[72] Cheeke PR. Actual and Potential Applications of Yucca Schidigera and Quillaja Saponaria Saponins in Human and Animal Nutrition. In: Saponins in Food, Feedstuffs and Medicinal Plants. Springer Netherlands; 2000. p. 241–54.

[73] EA T, D R, HF E. Dietary inulin lowers plasma cholesterol and triacylglycerol and alters biliary bile acid profile in hamsters. The Journal of nutrition [Internet]. 1998 [cited 2021 Sep 15];128(11):1937–43. Available from: https://pubmed.ncbi.nlm.nih.gov/9808646/. doi: 10.1093/jn/128.11.1937 9808646

[74] M B, C H, CG G. Bile salt hydrolase activity in probiotics. Applied and environmental microbiology [Internet]. 2006 Mar [cited 2021 Sep 15];72(3):1729–38. Available from: https://pubmed.ncbi.nlm.nih.gov/16517616/. doi: 10.1128/AEM.72.3.1729-1738.2006 16517616 PMC1393245

[75] Kockx M, Kritharides L. Intestinal cholesterol absorption. Current opinion in lipidology [Internet]. 2018 Dec 1 [cited 2021 Oct 3];29(6):484–5. Available from: https://journals.lww.com/co-lipidology/Fulltext/2018/12000/Intestinal_cholesterol_absorption.10.aspx. doi: 10.1097/MOL.0000000000000558 30239352

[76] EL M, HM F, AW B. Gut instincts: explorations in intestinal physiology and drug delivery. International journal of pharmaceutics [Internet]. 2008 Dec 8 [cited 2021 Oct 3];364(2):213–26. Available from: https://pubmed.ncbi.nlm.nih.gov/18602774/. doi: 10.1016/j.ijpharm.2008.05.012 18602774

