# Supplementary material for: Hypolipidemic activity of lactic acid bacteria: Adjunct therapy for potential probiotics
Source: PLoS One. 2022 Jun 23;17(6):e0269953. doi: 10.1371/journal.pone.0269953 (PMC9223303; doi:10.1371/journal.pone.0269953)
Supplement: S1 File — (PDF) [file pone.0269953.s008.pdf]

Table 1: API 50 CH fermentation patterns of the six selected LAB isolates.

	Isolate no.					
**Test substance**	**17**	**33**	**1**	**34**	**35**	**16**
**Control**	-	-	-	-	-	-
**Glycerol**	-	-	-	-	-	-
**Erythritol**	-	-	-	-	-	-
**D-Arabinose**	-	-	-	-	-	-
**L-Arabinose**	-	-	+	+	-	+
**D-Ribose**	+	-	+	-	+	+/-
**D-Xylose**	-	-	-	-	-	-
**L-Xylose**	-	-	-	-	-	-
**D-Adonitol**	-	-	-	-	-	-
**Methyl-D-xylopyranoside**	-	-	-	-	-	-
**D-Galactose**	+	-	+	+	+	+
**D-Glucose**	+	+	+	+	+	+
**D-Fructose**	+	+	+	+	+	+
**D-Mannose**	+	-	+	+	+	+
**L-Sorbose**	-	-	-	-	-	-
**L-Rhamnose**	-	-	-	-	-	-
**Dulcitol**	-	-	-	-	-	-
**Inositol**	-	-	-	-	-	-
**D-Mannitol**	-	-	+	+/-	-	+
**D-Sorbitol**	-	-	-	-	-	-
**Methyl-D-mannopyranoside**	-	-	-	-	-	-
**Methyl-D-glucopyranoside**	-	-	-	-	-	-
**N-acetylglucosamine**	+	+	+	+	+	+
**Amygdaline**	-	-	-	-	-	-
**Arbutine**	+	-	+	+	+	+
**Esculin**	+	+	+	+	+	+
**Salicin**	+	-	+	+	+	+
**D-Celiobiose**	+	+	+	+	+	+

**D-Maltose**	+	+	+	+	+	+
**D-Lactose**	+	+	+	+	+	+
**D-Melibiose**	-	-	+	+	-	+
**D-Saccharose**	-	+	+	+	-	+
**D-Trehalose**	+	-	+	+	+	-
**Inulin**	-	-	-	-	-	-
**D-Melezitose**	-	-	-	-	-	-
**D-Raffinose**	-	-	-	-	-	-
**Amidon**	-	-	-	-	-	-
**Glycogen**	-	-	-	-	-	-
**Xylitol**	-	-	-	-	-	-
**Gentiobiose**	+	-	+	+	-	+
**D-Turanose**	-	-	-	-	-	-
**D-Lyxose**	-	-	-	-	-	-
**D-Tagatose**	+	+	+	+	+	-
**D-Fucose**	-	-	-	-	-	-
**L-Fucose**	-	-	-	-	-	-
**D-Arabitol**	-	-	-	-	-	-
**L-Arabitol**	-	-	-	-	-	-
**Potassium gluconate**	-	-	-	-	-	-
**2-Keto-gluconate**	-	-	-	-	-	-
**5-Keto-gluconate**	-	-	-	-	-	-

(+) positive; (+/-) weakly positive; (–) negative.

API 50 CH STRIPS

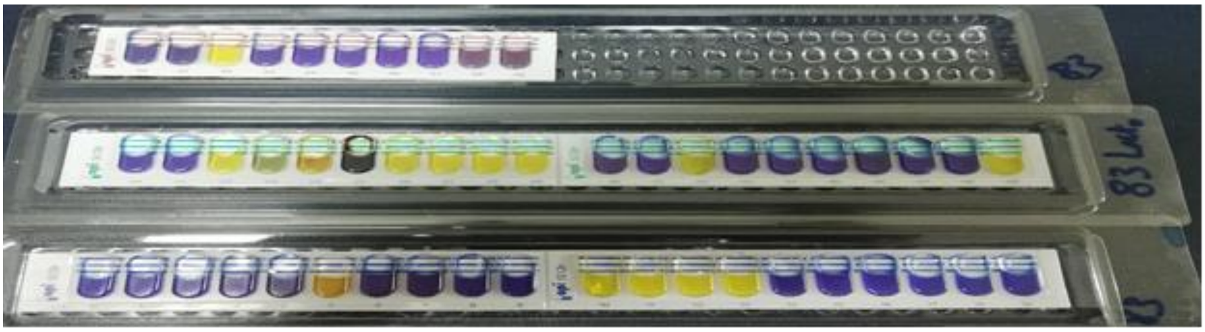

Figure 1 Isolate no. 17 (code= 83)

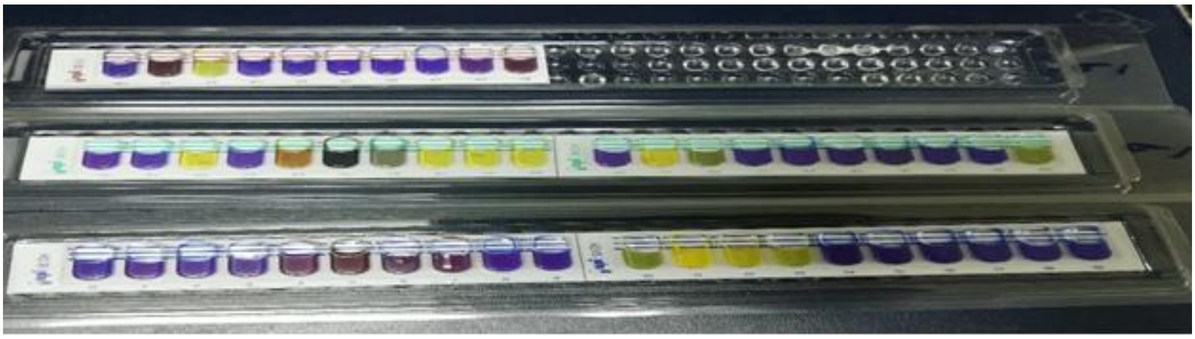

Figure 2 Isolate no. 33 (code= 9)

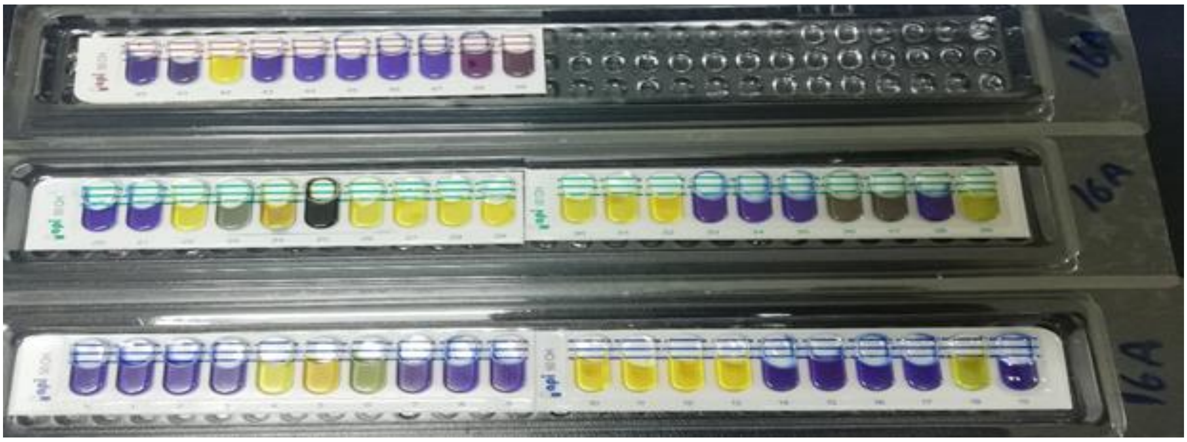

Figure 3 Isolate no.1 (Code= 16A)

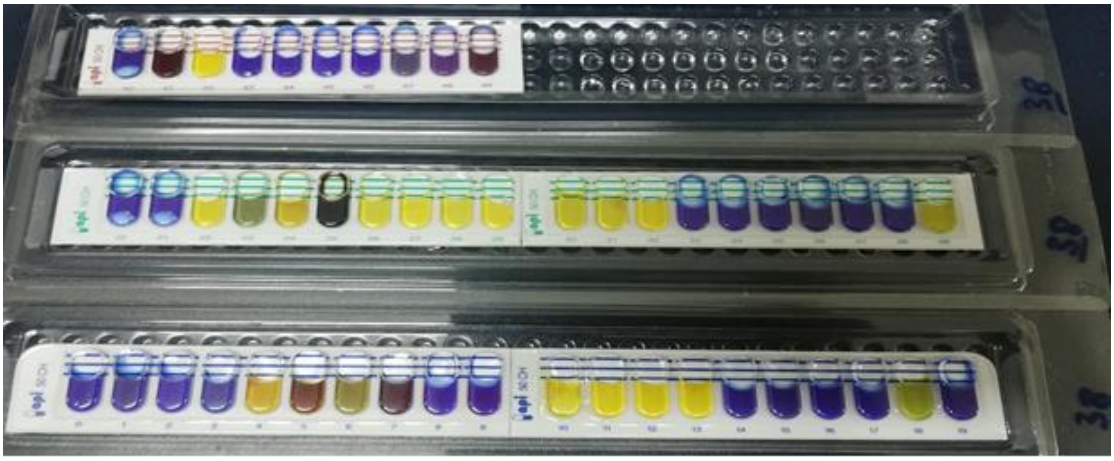

Figure 4 Isolate no. 34 (Code= 38)

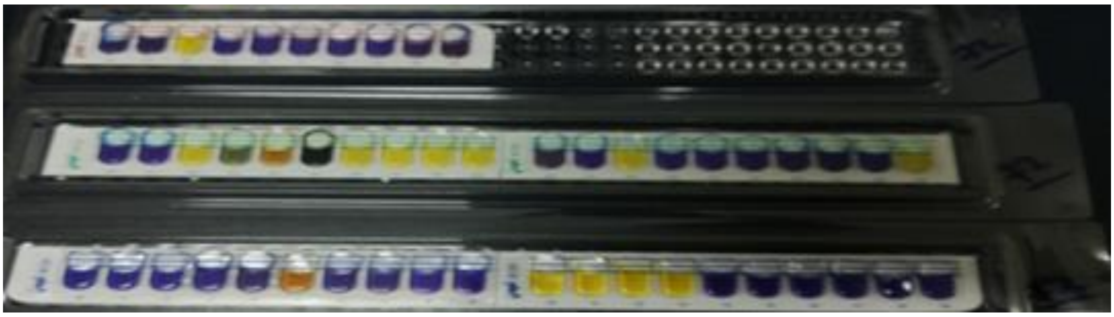

Figure 5 Isolate no. 35(Code= 72)

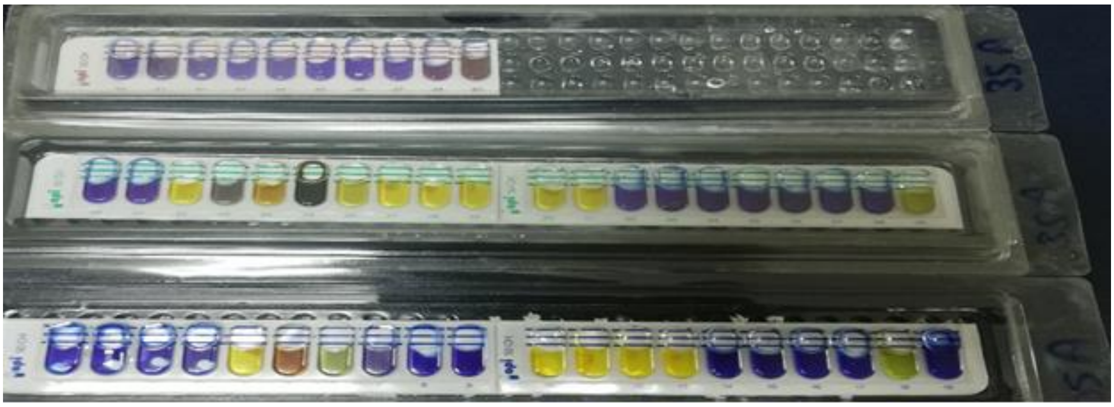

Figure 6 Isolate no.16 (Code= 35A)
